# Supplementary material for: Low threshold lasing emissions from a single upconversion nanocrystal
Source: Nat Commun. 2020 Dec 1;11:6156. doi: 10.1038/s41467-020-19797-4 (PMC7708641; doi:10.1038/s41467-020-19797-4)
Supplement: Supplementary file 1 — Supplementary Information [file 41467_2020_19797_MOESM1_ESM.pdf]

Supplementary Information

Low threshold lasing emissions from a single upconversion nanocrystal

Shang et al.

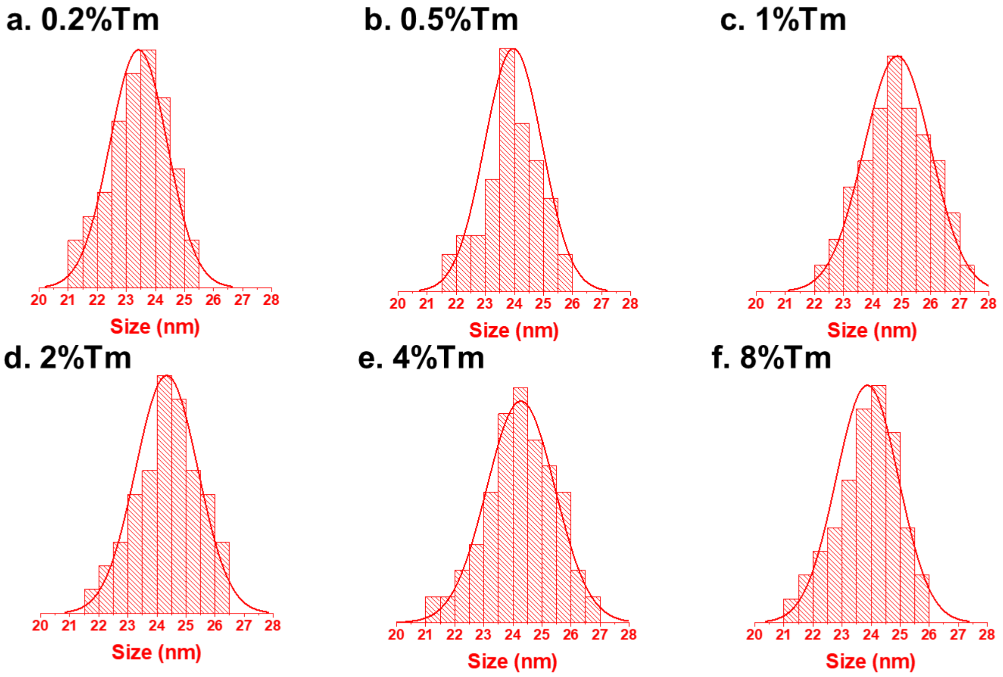

Supplementary Figure 1 Size distribution of the 24 nm UCNPs with different Tm^{3+} doping concentrations.

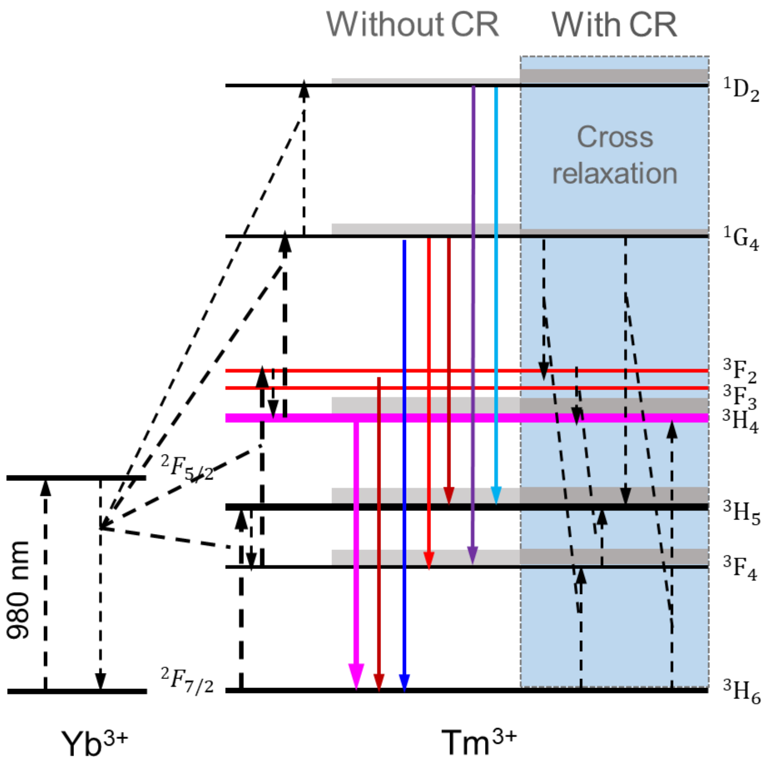

Supplementary Figure 2 Energy level diagram of Yb^{3+}/Tm^{3+} co-doped UCNPs including typical cross-relaxation pathways among Tm^{3+} emitters.

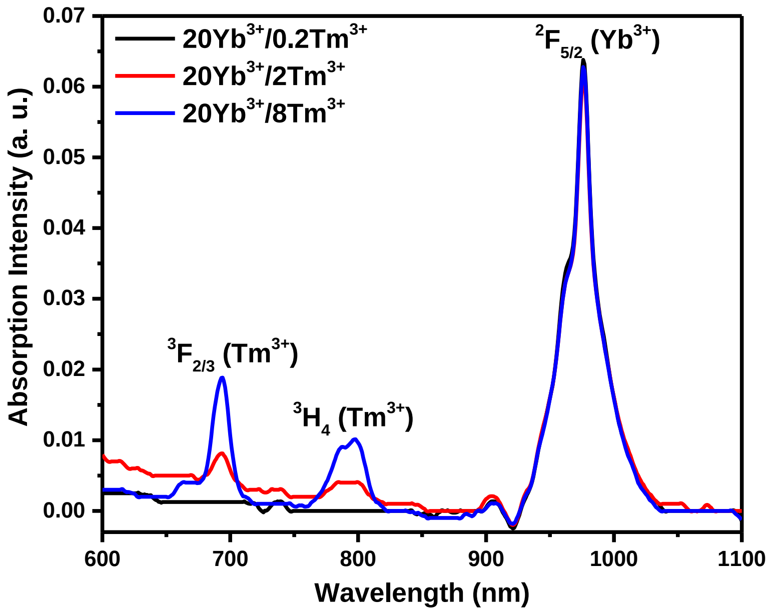

Supplementary Figure 3 Absorption spectra of NaYF4:20Yb^{3+}/$x$$Tm^{3+}$ (x=0.2, 2, 8).

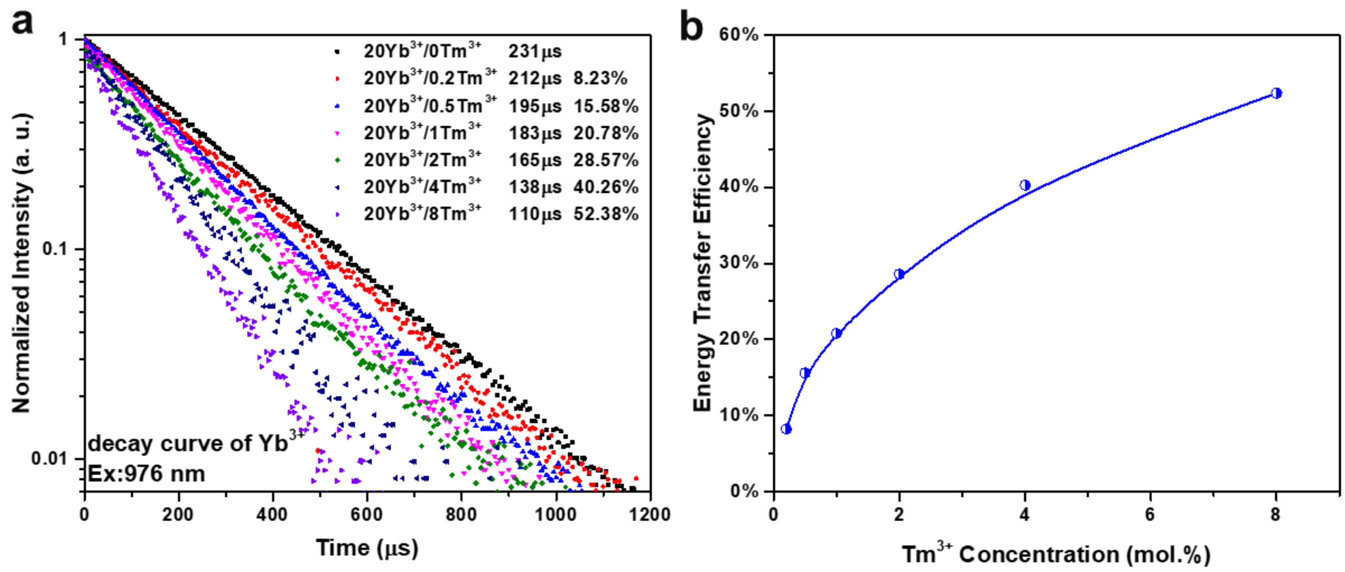

Supplementary Figure 4 a, Lifetime decay curves of Yb^{3+} emissions at 980 nm and **b**, Yb^{3+} to Tm^{3+} energy transfer efficiencies for UCNPs with different Tm^{3+} doping concentrations.

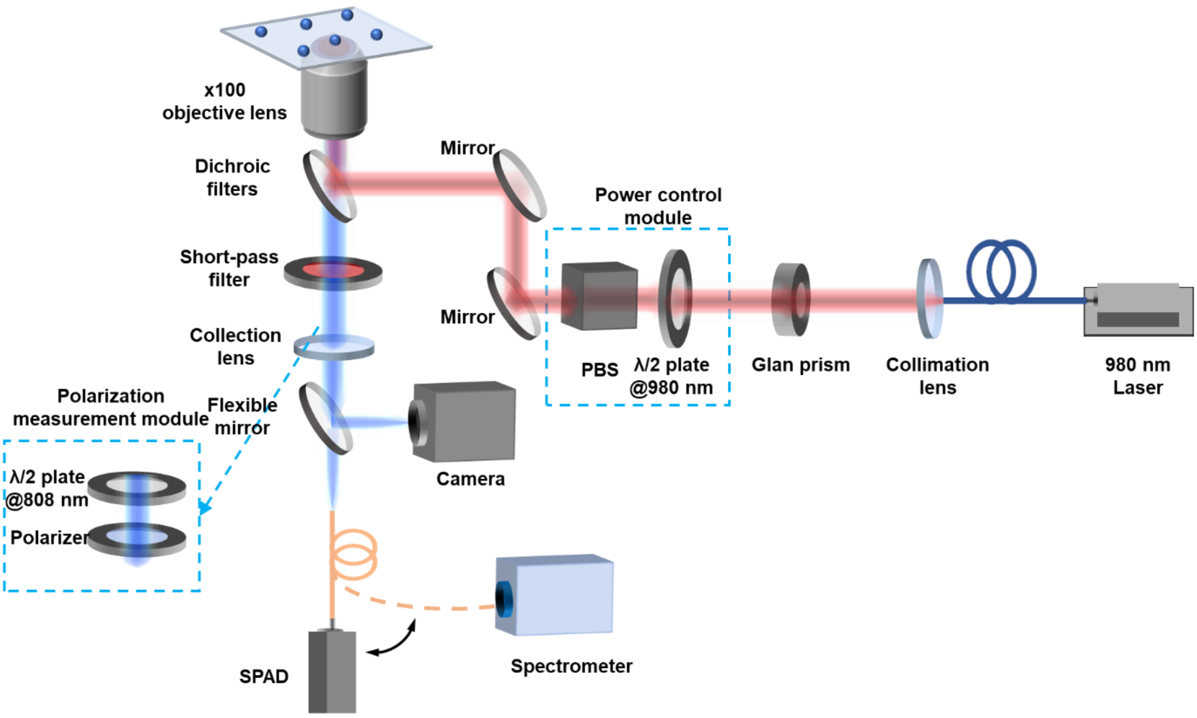

Supplementary Figure 5 Experimental setup for homemade confocal microscopy (the motor controlled rotatable half-wave plate is used to continuously adjust the excitation power; polarization measurement module was set up only to measure the polarization spectra; Camera, Prime 95B sCMOS and Nikon DS Fi1; SPAD, single-photon avalanche diode).

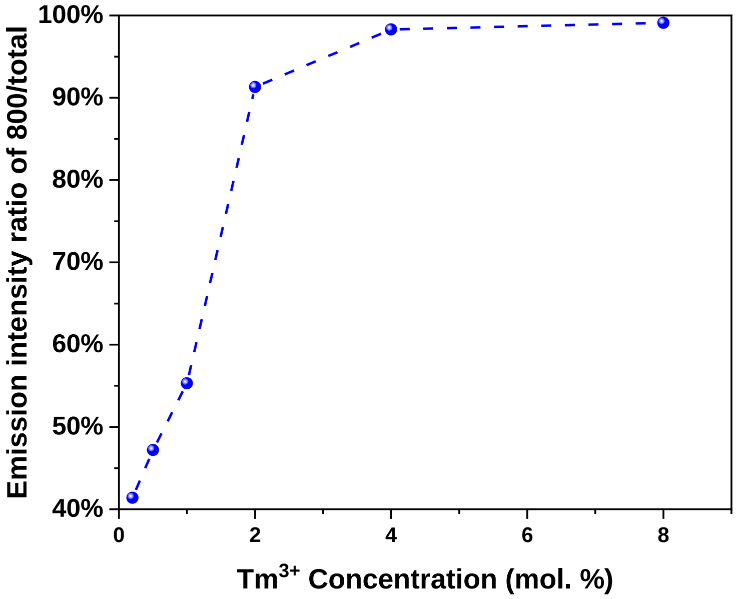

Supplementary Figure 6 The emission intensity ratio of 800 nm peak/total emission under the excitation of 100 W/cm^2.

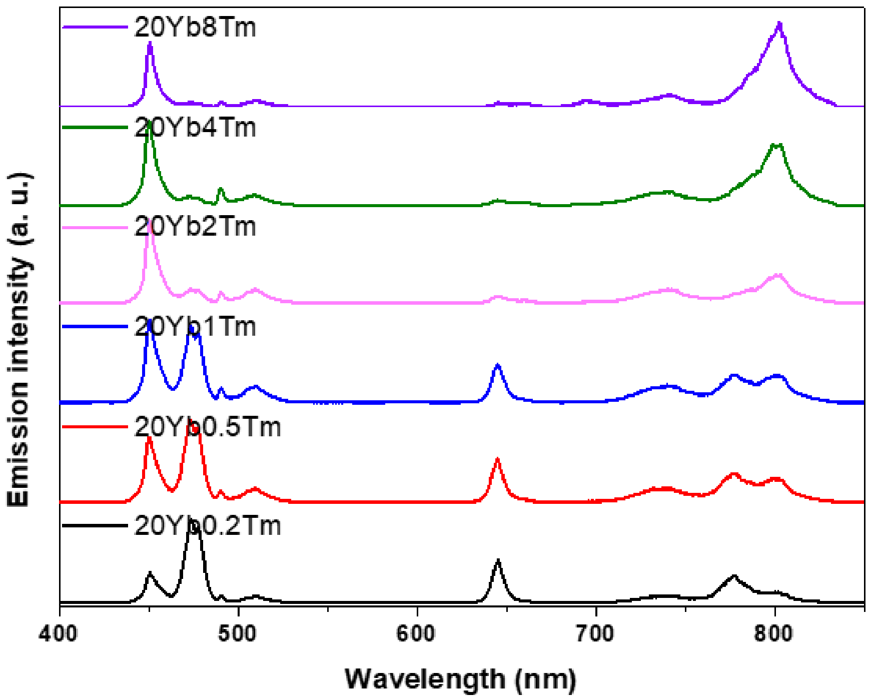

Supplementary Figure 7 Emission spectra of upconversion nanoparticles with various Tm^{3+} doping concentrations under the excitation of 100 kW/cm^2. Noting that, the emission distribution of different transition processes indicates the existence of CR.

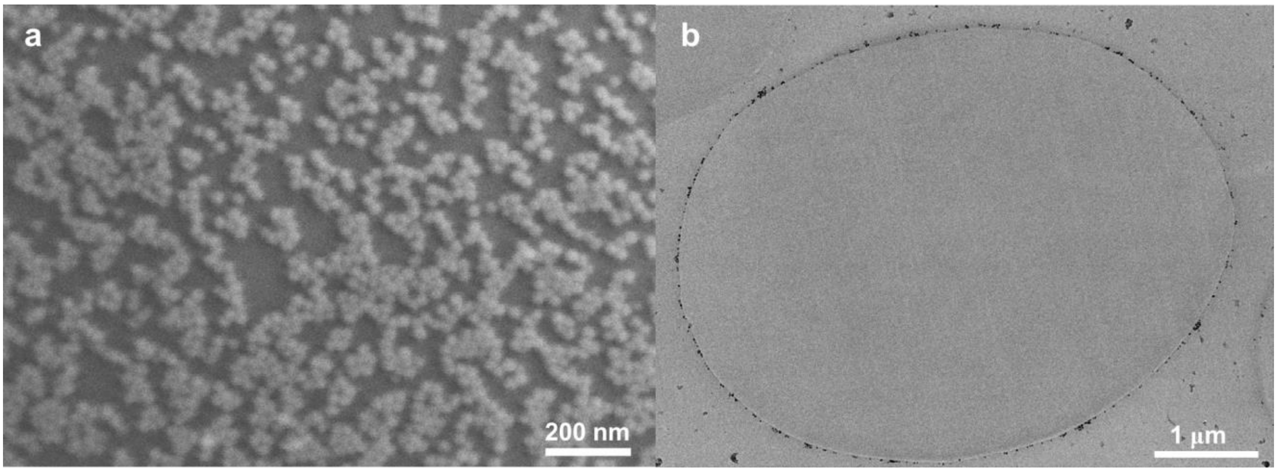

Supplementary Figure 8 a, Enlarged SEM image of microcavity surface; **b**, TEM image of epoxy embedded microtome cross -section (slice thickness: ~50 nm) of microcavity coated with a single layer of UCNPs. All the UCNPs are coated on the outer surface of the microsphere.

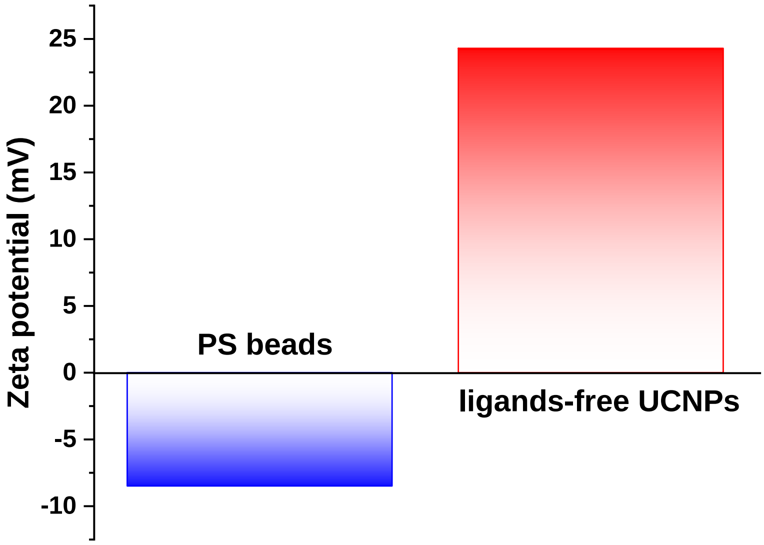

Supplementary Figure 9 Zeta potentials of 5 μm PS beads and ligands-free UCNPs.

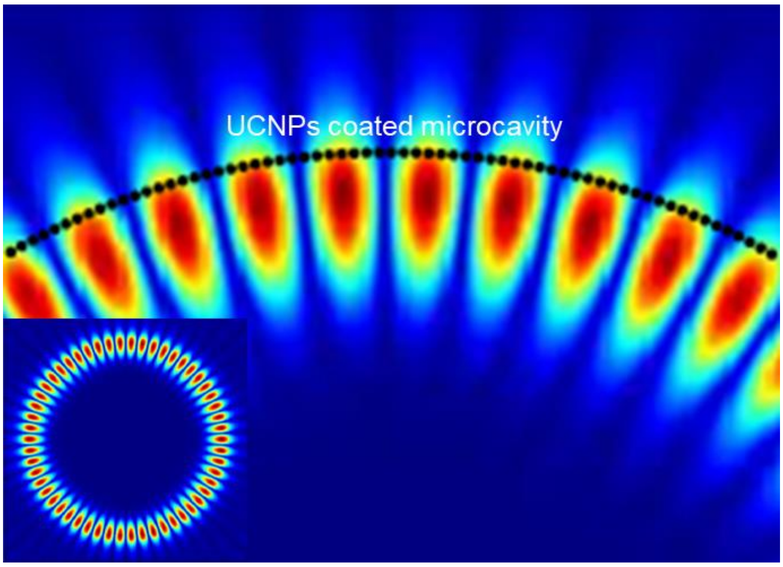

Supplementary Figure 10 Numerical simulation of the electrical-field distributions at 800 nm (eigenmode) within a major plane. Note that the black spots indicate upconversion nanoparticles coated on the surface of microcavity.

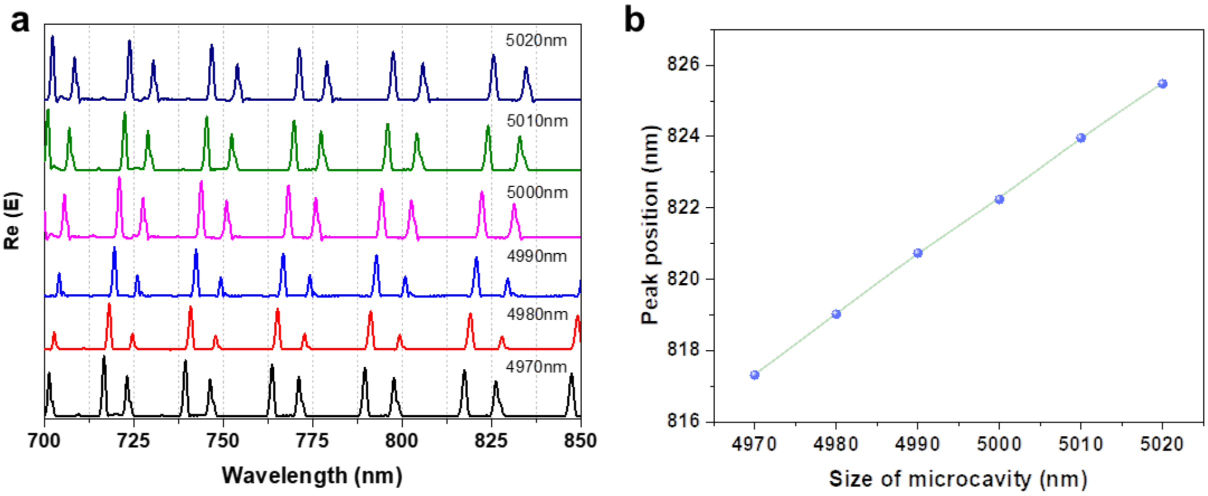

Supplementary Figure 11 **a**, Numerical simulations of resonance spectra of microcavity with different sizes. **b**, The plot of mode position *v.s.* cavity size.

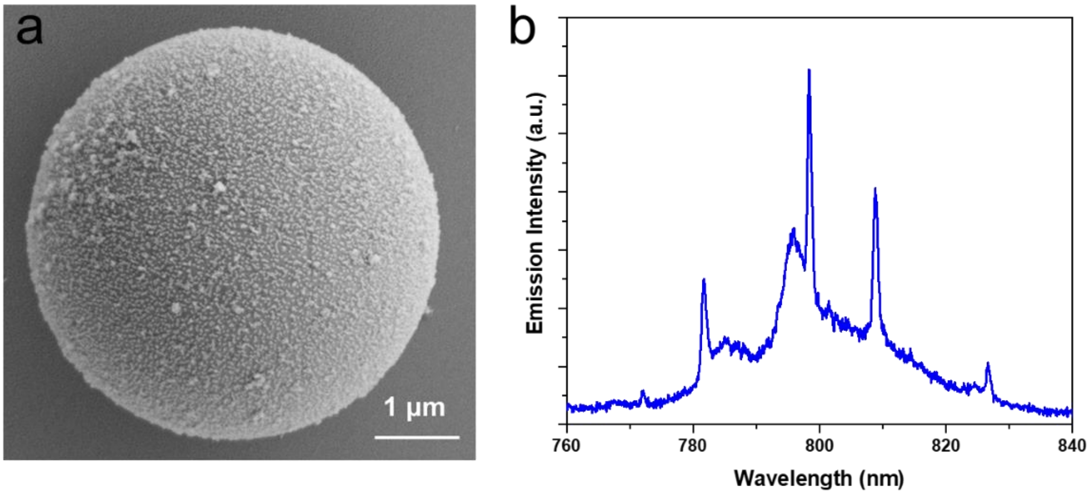

Supplementary Figure 12 **a**, SEM image and **b**, upconversion spectrum under the excitation of 980 nm laser (100 kW/cm^2) of the microcavity coated with 24 nm UCNPs. Aggregated nanoparticles at the surface induce the scattering cavity losses, in which the corresponding spectrum shows a strong background of spontaneous emission and increased FWHM of ~1.6 nm.

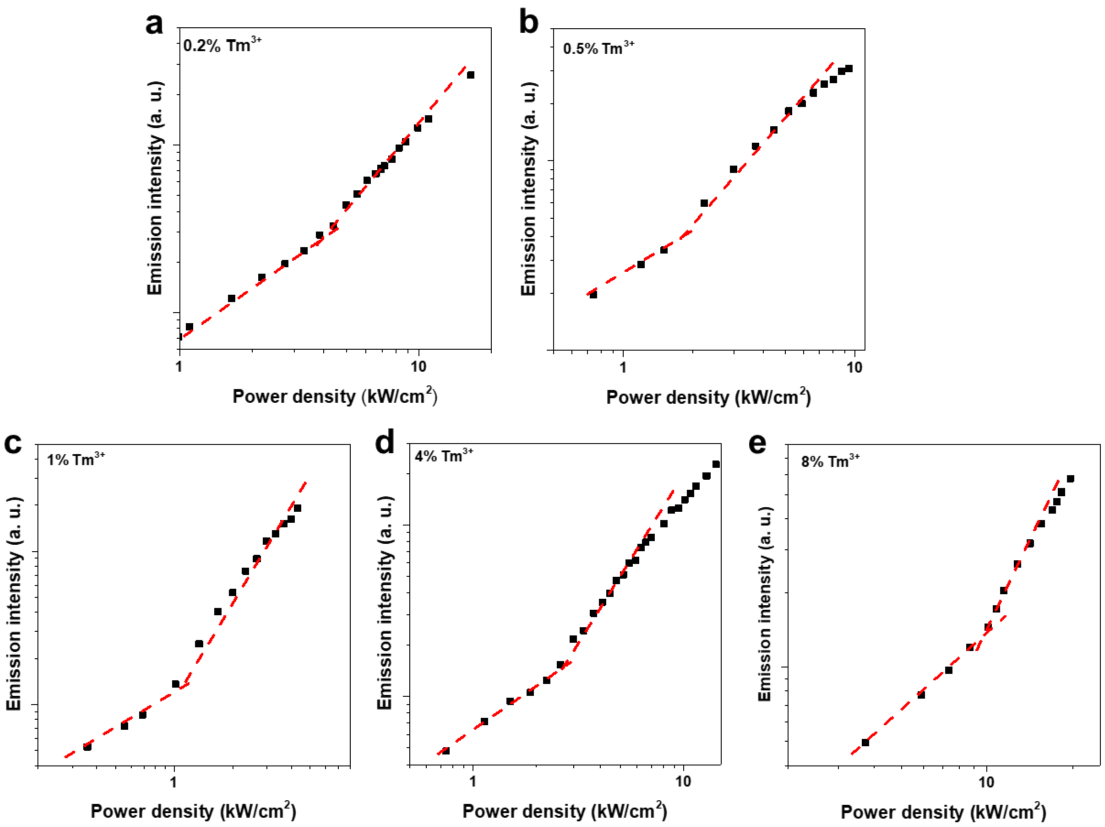

Supplementary Figure 13 The systematic characterizations of the lasing threshold for the cavities coated with UCNPs at Tm^{3+} doping of **a-e**, 0.2%, 0.5%, 1%, 4%, 8%.

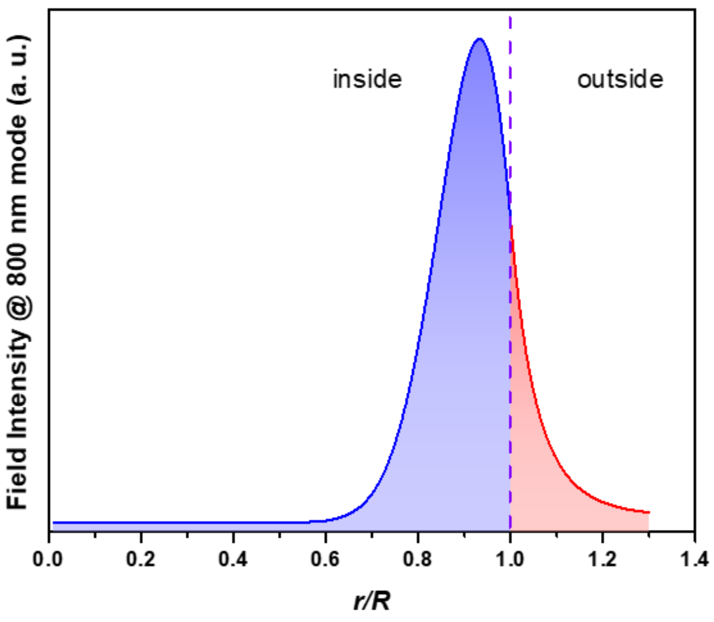

Supplementary Figure 14 The electrical-field distributions at 800 nm *v.s.* the distance to the center.
